# Understanding the genetic basis of blueberry postharvest traits to define better breeding strategies

**DOI:** 10.1093/g3journal/jkae163

**Published:** 2024-07-25

**Authors:** Gonzalo Casorzo, Luis Felipe Ferrão, Paul Adunola, Estefania Tavares Flores, Camila Azevedo, Rodrigo Amadeu, Patricio R Munoz

**Affiliations:** Horticultural Sciences Department, University of Florida, Gainesville, FL 32608, USA; Horticultural Sciences Department, University of Florida, Gainesville, FL 32608, USA; Horticultural Sciences Department, University of Florida, Gainesville, FL 32608, USA; Horticultural Sciences Department, University of Florida, Gainesville, FL 32608, USA; Department of Statistics, Federal University of Viçosa, Viçosa 36570, Brazil; Horticultural Sciences Department, University of Florida, Gainesville, FL 32608, USA; Bayer US—Crop Science, Chesterfield, MO 63017, USA; Horticultural Sciences Department, University of Florida, Gainesville, FL 32608, USA

**Keywords:** quantitative genetics, plant breeding, postharvest, blueberry, firmness, softening, Genomic Prediction, repeated measures, mixed models, heritability, fruit quality, GenPred, Shared Data Resources

## Abstract

Blueberry (*Vaccinium* spp.) is among the most-consumed soft fruit and has been recognized as an important source of health-promoting compounds. Highly perishable and susceptible to rapid spoilage due to fruit softening and decay during postharvest storage, modern breeding programs are looking to maximize the quality and extend the market life of fresh blueberries. However, it is uncertain how genetically controlled postharvest quality traits are in blueberries. This study aimed to investigate the prediction ability and the genetic basis of the main fruit quality traits affected during blueberry postharvest to create breeding strategies for developing cultivars with an extended shelf life. To achieve this goal, we carried out target genotyping in a breeding population of 588 individuals and evaluated several fruit quality traits after 1 day, 1 week, 3 weeks, and 7 weeks of postharvest storage at 1°C. Using longitudinal genome-based methods, we estimated genetic parameters and predicted unobserved phenotypes. Our results showed large diversity, moderate heritability, and consistent predictive accuracies along the postharvest storage for most of the traits. Regarding the fruit quality, firmness showed the largest variation during postharvest storage, with a surprising number of genotypes maintaining or increasing their firmness, even after 7 weeks of cold storage. Our results suggest that we can effectively improve the blueberry postharvest quality through breeding and use genomic prediction to maximize the genetic gains in the long term. We also emphasize the potential of using longitudinal genomic prediction models to predict the fruit quality at extended postharvest periods by integrating known phenotypic data from harvest.

## Introduction

Blueberry (*Vaccinium* spp.) is among the most-consumed soft fruit and has been recognized as an important source of health-promoting compounds (Kalt *et al*. 2020). In the past 20 years, its production has increased significantly, mainly due to the expansion in consumption and producers’ profitability (Brazelton *et al*. 2022). As a consequence, new producers are entering this business, causing tighter competition and new demands for plant breeding programs. While most breeding programs have historically focused on developing cultivars with high yield and fruit quality attributes, the current global demand requires a new group of cultivars that can be shipped long distances and stored for several weeks before they become available for consumers.

Being perishable and susceptible to rapid spoilage, the postharvest quality is a key component for the blueberry market. Currently, not many cultivars can maintain their fruit condition and quality to satisfy the demands of distant markets. Among the important traits required by growers and retailers to maintain the postharvest quality, fruit firmness is one of the most important (Gilbert *et al*. 2015). During storage, firmness can decline due to cell wall degradation (Chen *et al*. 2015), dehydration of the fruit (Paniagua *et al*. 2013), or physical damage during harvest and transport operations (Moggia *et al*. 2017). Another key trait impacting consumer experience is flavor. Mainly dictated by the concentration of sugar and acids, recent studies indicate that flavor profile could change during postharvest storage depending on the genotype (Perkins-Veazie *et al*. 1995; Moggia *et al*. 2016; Retamales and Hancock 2018). This fact not only suggests that such variations may be genetically controlled but also sheds light on the relevance of breeding programs for expanding shelf life.

Among the multiple initiatives focused on improving the shelf life, plant breeding provides the best means to increase the phenotypic performance and release cultivars that could meet market demands. As an outcrossing and clonally propagated crop, blueberry breeding programs are conventionally organized in a recurrent selection scheme, in which population improvement and product development are incorporated into the same pipeline (Pott *et al*. 2020). In the traditional system, cultivar development can take 12–15 years, making breeding cycles costly and time-consuming (Lyrene 2008). During this process, plant breeders have relied on quantitative genetic methods to monitor the genetic gains, estimate the genetic parameters, and increase the mean phenotypic performance in selected populations (Bernardo 2020). The integrated use of pedigree information restricted maximum likelihood (REML) and best linear unbiased prediction (BLUP) has proven to be an efficient methodology for parental selection and design of future crosses (Munoz *et al*. 2014; Cellon *et al*. 2018).

Despite the efficiency of pedigree BLUP analyses, modern breeding programs are transitioning to genomic-assisted breeding, using molecular markers covering the entire genome to predict the genetic merits of individuals for complex traits. In blueberry, the methodology has been applied in the form of genomic selection for multiple fruit quality traits, with the potential to maximize genetic gains by shortening cycles and increasing selection accuracy (de Bem Oliveira *et al*. 2019; Amadeu *et al*. 2020; Ferrão *et al*. 2021). We hypothesize that this approach may be particularly attractive for postharvest traits, given the difficulty and costs associated with phenotyping large populations. In strawberry, for example, high predictability abilities have been recently reported for postharvest decay, showing the potential to incorporate genomic prediction for assisting in the selection of long-shelf life individuals into the scope and pace of fruit breeding programs (Petrasch *et al*. 2022). The importance of predicting blueberry shelf life has been recently addressed by Oh *et al*. (2024), who successfully trained prediction models using fruit quality and textural traits. However, no research has yet been published about implementing genomic prediction for postharvest traits.

Aiming to advance our understanding of the genetic basis of fruit quality traits that mainly affect the shelf life of blueberries, we combined genomic information and longitudinal mixed-model analyses to describe and predict the phenotypic variation of blueberry genotypes maintained at the University of Florida (UF) breeding program. With a long breeding history, UF has contributed significantly to expanding production areas by developing low-chilling cultivars. The so-called southern highbush blueberry (SHB) varieties represent almost 40% of the current global production and are better adapted to subtropical and mild Mediterranean climates (Brazelton *et al*. 2022). Given its importance in global consumption and production, we addressed the following questions: (1) Are the postharvest-related quality traits genetically controlled? (2) What is the current diversity of the SHB breeding population for postharvest variation? (3) Are certain traits and genotypes more subjected to variations during storage time points? (4) Can we use genomic information to predict these traits along the postharvest storage? (5) Can we leverage these predictions using longitudinal prediction models? We anticipate that understanding the genetic basis of traits subject to postharvest variation will lead us to develop breeding strategies for effectively selecting genotypes with exceptional postharvest quality and, consequently, releasing cultivars that combine high fruit quality and longer shelf life.

## Materials and methods

### Plant material and experimental design

In this study, we used 588 advanced-selection genotypes from the UF Blueberry Breeding Program. These genotypes come from 343 different families, resulting in a population with high genetic variation and no observed population structure. The harvest and evaluation of all the genotypes were carried out between April and June and sparse across 2021, 2022, and 2023. Sixteen genotypes were harvested and evaluated in every year to serve as experimental checks for estimating the year effect, following an augmented experimental design. More details about the blueberry breeding cycles are discussed by Cellon *et al*. (2018) and Ferrão *et al*. (2021).

In the field trials, genotypes were randomly arranged in plots of 15 clones without repetitions. Berries from each genotype were harvested using commercial standards directly into eight 4.4-oz clamshells (Pactiv A9756) when the fruit was fully ripe (100% blue color). All clamshell samples were stored in corrugated blueberry shipper boxes and placed on shelves inside a cooling room at 1°C. In the cooling room, we mimicked commercial operation by placing the samples on shelves and covering each shelf with a clear PVC cover to achieve a more uniform relative humidity among the samples. For the postharvest treatments, we selected 4 time points: 1 day (1D), 1 week (1W), 3 weeks (3W), and 7 weeks (7W). The reason for selecting these time points is based on different market targets. On average, local consumers will consume berries after 1W of cold storage, while distant markets will consume them after 3W and 7W depending on the distance. A different set of fruit of the same genotype was phenotyped after each time point. We used 2 technical repetitions for each time point, and the experiment in the cooling room was arranged as a completely randomized design. It is important to remark that the fruit of both repetitions came from the same experimental unit in the field; therefore, they were not considered as independent repetitions in the experimental design and were averaged prior to the statistical analysis.

### Fruit quality phenotyping

#### Fruit firmness and size

Fruit firmness was measured using a Fruit Firm 1000 machine (CVM, Inc.) after samples were warmed to room temperature (∼20°C). For each time point, a sample of 26 fruits per genotype was positioned on a rotating plate and compressed equatorially. Firmness results were calculated as the mean force to deflect the surface of the fruit by 1 mm (g/mm). Firmness differential (Δfirmness) was calculated as the difference in the compression force of each genotype between a given time point (*n* = 1W, 3W, 7W) and its 1D treatment (considered as our initial firmness). Fruit size was also measured by the Fruit Firm 1000 and expressed as fruit diameter (mm).

#### Water loss

Water loss percentage (WL) was measured as the weight loss percentage during postharvest storage. It was calculated using the following equation:


$$\mathrm{WL}(\text{\%})=(W_{i}-W_{f})/W_{i}\times 100,$$

where $W_{i}$ is the initial weight and $W_{f}$ is the final weight of the sample. A precision digital scale was used (Mettler Toledo, Inc.). $W_{i}$ was measured right before placing the samples in the cooling room and $W_{f}$ at the end of the storage period. Water loss was only measured at the 7W storage period.

#### Waxy bloom

The bloom is the outer layer of wax on blueberries. It gives a powdery light-blue appearance to the fruit. We used a visual score for the bloom to classify genotypes as 0–25%, 25–50%, 50–75%, and 75–100% surface coverage. A single score was assigned to the entire clamshell at each time point.

#### Pedicel scar size

To determine the pedicel scar size, a subsample of 10 berries was visually categorized as small, medium, or large scars. The scar size coefficient was calculated as follows:


Scar(1Ns+2Nm+3Nl)/(Ns+Nm+Nl),

where *Ns* is the number of berries with a small scar, *Nm* is the number of berries with a medium scar, and *Nl* is the number of berries with a large scar. Scar size was only phenotyped at the 1D time point.

#### Shriveling severity

Shriveling severity was evaluated using visual scores as follows: no apparent shriveling (0); slight wrinkles (1); marked wrinkles and creases (2); and berries starting to collapse (3). A single score was recorded for each clamshell at each time point.

#### Soluble solid content and total titratable acidity

Samples from each genotype and time point were also used for chemical analyses. To this end, a representative sample of each clamshell was selected, blended, and centrifuged. The obtained juice was evaluated for soluble solid content (SSC) and total titratable acidity (TTA). SSC and TTA were measured with a digital pocket refractometer (Atago USA, Inc.) and an automatic titrator (Mettler Toledo, Inc.).

#### Fungal decay

Fruit decay was assessed by quantifying the percentage of the fruit weight exhibiting signs of fungal proliferation within each clamshell.

### Plant genotyping

During the summer of 2021 and 2022, young leaf tissues were sampled from each individual within the breeding population. Genomic DNA was extracted and subsequently genotyped at RAPiDGenomics (Gainesville, FL, USA) by utilizing a sequence capture technique. The isolated DNA underwent sequencing on the Illumina HiSeq platform, employing 150-cycle paired-end runs aimed at 10,000 probe regions specifically curated for this sequence capture strategy. The obtained raw reads were subjected to quality-based filtering and trimming via TRIMMOMATIC v0.39. These refined reads were then aligned to the *Vaccinium corymbosum* cv ‘Draper’ reference genome (Colle *et al*. 2019). Single nucleotide polymorphisms (SNPs) were identified using the FreeBayes v.1.0.1 software (Garrison and Marth 2012). Given the tetraploid nature of the blueberry (2*n* = 4*X* = 48), allele dosages were determined based on read counts using the UPDOG R package (Gerard *et al*. 2018). After excluding SNPs with a significant degree of missing data (>80%), a total of 52,900 markers were retained for subsequent analyses. A plot of the first 3 principal components of the genomic matrix can be found in Supplementary Fig. 1.

### Phenotypic analysis

A longitudinal linear mixed model was fitted using all the postharvest time points and their interaction with the genotypic effect simultaneously. The model was fitted in the ASReml-R package (Butler *et al*. 2018) as follows:


$$y=X_{1}\beta +X_{2}t+X_{3}g_{1}+Zg_{2}+e,$$

where y is the vector of phenotypes at all time points, β is the vector of the population mean at each time point plus the year of evaluation, and ***t*** is a vector of fixed effects of the postharvest period. We split the genotypic effect into 2 components using a similar approach to that reported by Pastina *et al*. (2012) and de C. Lara *et al*. (2019), where $g_{1}$ is the vector of fixed effects of experimental checks connecting the years, and $g_{2}$ is the vector of random effects of regular genotypes. Finally, e is the vector of random residual effects. The Gaussian distribution was assumed for the genetics ($g_{2}$) and residual effects (e), $g_{2}∼\mathrm{MVN}(0,\sum_{t}⊗G)$ and $e∼\mathrm{MVN}(0,\sum_{e}⊗I)$, where ***G*** is the additive relationship matrix calculated with the molecular marker information and constructed using the VanRaden methodology assuming tetrasomic inheritance, as implemented in the AGHmatrix R package (Amadeu *et al*. 2016, 2023); and I is an identity matrix. The terms $\sum_{t}\;\;$ and $\sum_{e}\;\;$ are unstructured variance–correlation structures for the postharvest time effect and the residuals, respectively; while $X_{1}$, $X_{2}$, $X_{3}$, and ***Z*** are the incidence matrices. To assess the goodness of fit statistics, a simpler model assuming no correlation between time points or residuals was fitted and compared to the more complex longitudinal model using the Bayesian information criterion (BIC) (Neath and Cavanaugh 2012). The BIC was consistently lower for all traits when assuming an unstructured correlation in the model, indicating that the unstructured correlation model provides a better fit for the data (Supplementary Table 1). More details about the statistical models are presented in the Supplementary material.

Variance components, genetic correlations, and breeding values were computed using the REML/BLUP approach, as implemented in ASReml-R. The narrow-sense heritability of the *i*th time point ($h_{i}^{2}$) was estimated as follows:


$$h_{i}^{2}=\frac{\sigma_{g}^{2(i)}}{\sigma_{g}^{2(i)}+\sigma_{e}^{2(i)}},$$

where $\sigma_{g}^{2(i)}$ and $\sigma_{g}^{2(j)}$ are the genetic variances of the *i*th and *j*th time points, respectively. Correlations between traits were computed as Pearson's correlation between the phenotypic values of the traits. The significance of the postharvest period effect was evaluated for each trait using the Wald test. The significance of the genotype-by-time interaction was assessed using a likelihood ratio test between the complete model (considering the interaction term) and the reduced model (without accounting for the interaction effect). For both tests, a significance threshold level of 0.05 was used.

Additionally, we quantified the proportion of crossover interactions using a similar approach to the one reported by Van Eeuwijk *et al*. (2016). The post hoc test consists of evaluation pairs of genotypes, *i* and $i^{\prime }$, and pairs of environments (time points), *j* and $j^{\prime }$, for which either of the conditions $(y_{ij}-y_{i^{\prime }j})>0$ and $(y_{i{\;j}^{\prime }}-y_{i^{\prime }{\;j}^{\prime }})<0$ is satisfied or $(y_{ij}-y_{i^{\prime }j})<0$ and $(y_{i{\;j}^{\prime }}-y_{i^{\prime }{\;j}^{\prime }})>0$. This was achieved by randomly sampling pairs of genotypes at each pair of time points. When the BLUPs of 1 genotype were consistently larger than those of the other at both time points, it indicated no crossover interaction. This process was repeated over 100,000 iterations for each pair of time points, and the proportion of crossover events to total events was reported.

### Genomic prediction

Genomic prediction models were fitted using the ASReml-R package, following the GBLUP methodology (VanRaden 2008). A longitudinal model similar to the reported for the phenotypic analysis was used for genomic prediction, assuming unstructured variance and covariance matrix connecting time points for the interaction term and residuals. A genomic relationship matrix (***G*** matrix) was used to account for the correlation between genotypes, as described by Ferrão *et al*. (2021). A 10-fold cross-validation was performed to compute the model's prediction ability (PA). This approach involved using 90% of the individuals as a training population to predict the GEBVs of the remaining 10%. Individuals were randomly sampled into the training or testing population, and 10 iterations were performed to calculate the mean PA of each model. PA was represented as the Pearson's correlation between the phenotypic values and the genomic estimated breeding values (GEBVs). Four different cross-validation scenarios were tested: the so-called CV1 approach, in which the model predicted genotypes without phenotypic data at any time point; and 3 CV2 scenarios where genotypes at 7W postharvest were predicted, knowing their phenotype at different earlier time points (1D, 1W, or 3W). A visual aid of the cross-validation schemes can be found in Supplementary Fig. 2.

## Results and discussion

### Phenotypic distribution

**Fig. 1.**
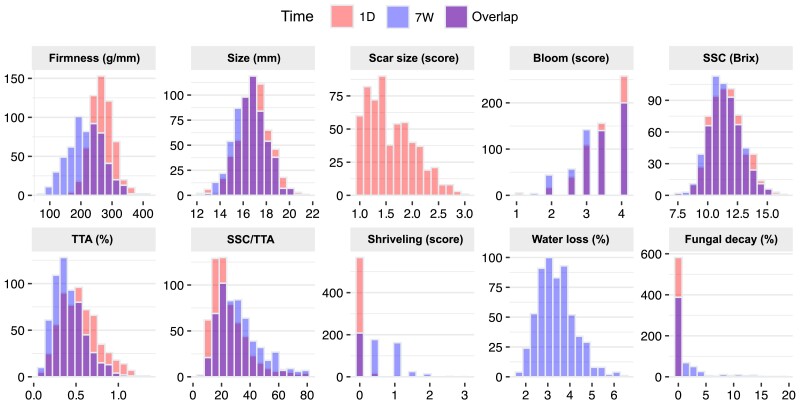
Phenotypic distribution of SHB breeding population fruit quality traits after 1D and 7W of postharvest storage at 1°C. Each value corresponds to a different genotype.

In this study, 10 fruit quality traits were measured over 4 time points in 588 SHB genotypes. For most of the traits, we observed a considerable phenotypic variation between values collected at 1D and 7W of storage (Fig. 1). Only fungal decay and shriveling showed a low phenotypic variation both at harvest and after postharvest storage. We hypothesize that the low variability is associated with our harvest and postharvest conditions, as they were designed to meet commercial standards. Consequently, there were no favorable conditions for these traits to fully express. Bloom scores were variable among genotypes and followed a left-skewed distribution.

Fruit firmness and TTA were the traits that showed higher variations across the postharvest time points. The population mean for firmness increased by 13 g/mm after 1W and 4 g/mm after 3W of postharvest storage, compared to the 1D treatment. After 7W of storage, the mean firmness was 51 g/mm lower than the 1D treatment, indicating that the softening occurred mainly between 3W and 7W. Also relevant, the maximum firmness recorded after 7W was 416 g/mm, suggesting that not all the genotypes were softening at the same rate. The mean TTA decreased linearly along the postharvest period, reaching an average of 0.42% at 7W (a difference of 0.12% compared to the 1D time point). Mean fruit size, bloom, and SSC changed in a very small magnitude during the postharvest treatment. Postharvest variations were statistically significant for all traits, while the genotype-by-time interaction was significant in all traits but SSC (Table 1).

**Table 1.** Genetic parameters computed using longitudinal mixed models. Narrow-sense heritability (*h*^2^) of the blueberry fruit quality traits after 4 postharvest storage time points at 1°C (1D, 1W, 3W, and 7W).

Trait	*h* ^2^_1d_	*h* ^2^_1w_	*h* ^2^_3w_	*h* ^2^_7w_	LRT *P*-value	*Ψ*_1d–7w_
Firmness (g/mm)	0.54	0.49	0.50	0.57	<0.001	0.25
TTA (%)	0.57	0.51	0.53	0.48	<0.001	0.03
SSC (brix)	0.36	0.39	0.38	0.44	0.104	0.03
Size (mm)	0.42	0.43	0.39	0.36	0.042	0.07
Bloom (visual score)	0.29	0.26	0.26	0.27	0.003	0.13
ΔFirmness (g/mm)*^a^*	—	0.29	0.35	0.45	<0.001	0.20
ΔTTA (%)*^a^*	—	0.00	0.00	0.10	0.002	0.01
Shriveling (visual score)	—	0.06	0.10	0.26	<0.001	—
Water loss (%)	—	—	—	0.18	—	—
Scar size (visual score)	0.43	—	—	—	—	—

Likelihood ratio test (LRT) *P*-values below 0.05 suggest a significant genetic-by-time interaction. Proportion of the crossover genetic-by-time interactions shows breeding values between 1D and 7W of storage (*Ψ*_1d–7w_). The genetic and residual variance components are displayed in Supplementary Table 2. The crossover interactions between different pairs of time points can be observed in Supplementary Table 3.

^*a*^Because these traits did not have data at 1D, the displayed value corresponds to *Ψ*_1w–7w._

### Fruit firmness and TTA varied the most during postharvest storage

**Fig. 2.**
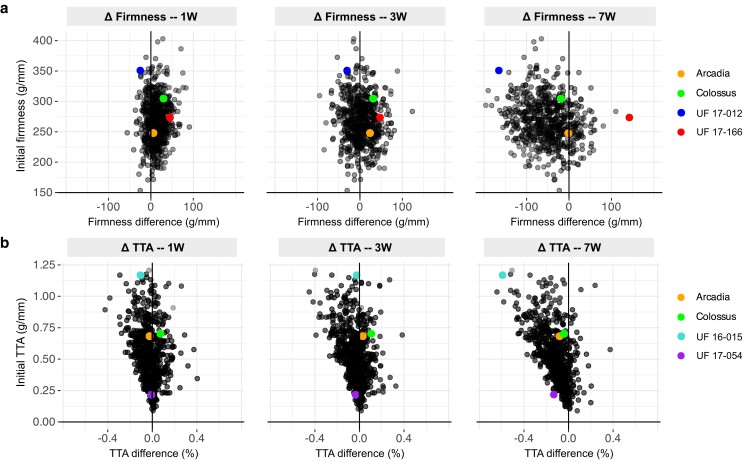
Fruit firmness a) and TTA b) variation of the breeding population after 1W, 3W, and 7W of postharvest storage at 1°C. Each point corresponds to a different genotype. Well-known cultivars and selections showing contrasting behaviors were highlighted.

Among the traits, fruit firmness showed the largest phenotypic variation along the time points studied. Linear regressions were performed, and the coefficient of determination (*R*^2^) was recorded to determine to what extent the variance in fruit firmness at harvest could explain the firmness after different postharvest storage periods (Supplementary Fig. 3). Initial firmness could explain 79% and 58% of the firmness at 1W and 3W, respectively; however, it could only explain 23% of the final firmness at 7W (*P* < 0.001). In short, our results suggest that a firm genotype at harvest will likely remain firm after 1W and 3W of cold storage, indicating that initial firmness is an important metric for predicting shorter storage periods. This result is particularly important for selections targeting local markets where blueberries are commercialized after a few weeks of storage. In contrast, lower values were observed after 7W storage, indicating that firmness at harvest, by itself, is not a good selection index for long storage periods. Losses of firmness during postharvest are consistent with other blueberry studies that reported similar trends (Giongo *et al*. 2013; Moggia *et al*. 2016). When differences in firmness (Δfirmness) were quantified, we noticed considerable variation among genotypes (Fig. 2a). For example, after 1W, 24% of the genotypes were softer compared to their initial firmness. This proportion was substantially higher after 3 W and 7W, with 45% and 84% of the genotypes showing reductions, respectively. Simultaneously, our results indicate that 16% of the population analyzed had higher firmness values after 7W of storage compared to their initial firmness.

Loss of firmness is a common issue during postharvest in fruits and vegetables. Different biological reasons can explain blueberry softening, including the effect of cell wall degradation (Deng *et al*. 2014; Chen *et al*. 2015), dehydration of the fruit (Paniagua *et al*. 2013), or physical damage during harvest and postharvest operations (Moggia *et al*. 2017). Fruit softening during postharvest storage is a common phenomenon in fruit crops, such as apples (Johnston *et al*. 2002), grapes (Ejsmentewicz *et al*. 2015), and tomatoes (De Ketelaere *et al*. 2004). Paradoxically, while loss of firmness is a well-reported phenomenon, we observed an oppositive trend with some genotypes increasing their firmness during the storage time. An extreme example was the genotype “UF 17–166,” which firmness increased 142 g/mm during the 7W of postharvest storage. From our knowledge, no article in the blueberry literature has shown consistent increases in firmness during postharvest using such a large and diverse genotypic panel. Similar studies also reported increments in fruit firmness after 6 weeks and 8 weeks of cold storage, but with some important considerations (Giongo *et al*. 2013; Farneti *et al*. 2020). The authors interpreted the results as an excessive elasticity created by the loss of internal water turgor pressure in the berry, which enhanced the resistance to the texture analyzer's probe penetration. A microstructural analysis suggested that the postharvest firming of some blueberry genotypes might also be caused by the number and arrangement of flesh stone cells and their cell wall thickness (Allan-Wojtas *et al*. 2001). Remarkably, we also noticed a high overall liking associated with firmness increment (results not reported), which opened important venues for developing future cultivars that aggregate postharvest and flavor quality. Importantly, these genotypes offer a valuable opportunity to further study the impact of postharvest storage on blueberry flavor in future experiments.

Another fruit quality trait that changed considerably during postharvest storage was the TTA (Fig. 2b). After 7W, the TTA of 23% of the individuals decreased by more than 0.2. We observed greater TTA reductions in genotypes with a higher TTA at harvest. Reductions in the TTA during postharvest storage have been previously reported in blueberries; however, these changes have not been observed consistently among experiments and genotypes (Perkins-Veazie *et al*. 1995; Chiabrando *et al*. 2009; Chiabrando and Giacalone 2017). In other fruit crops, a decrease in acidity is typical during postharvest storage, and it has been attributed to the use of organic acids as substrates for respiratory metabolism (Al-Dairi *et al*. 2021). However, their concentration may also increase due to high water loss rates (Echeverría *et al*. 2009), indicating that storage conditions can impact the trend of the TTA during storage. In our study, we did not find any correlation between changes in the TTA and water loss (*r* = 0.04).

### Fruit traits associated with postharvest quality can be genetically improved

Understanding the genetic architecture of complex traits is a crucial step to improve postharvest traits through breeding. Breeders have guided their decisions based on the level of genetic control (i.e. heritability), magnitude of gene action effects, correlations between traits, and dynamics of genotype-by-environment interactions (Bernardo 2020). A key parameter is narrow-sense heritability (*h*^2^), which has a genetic source and a direct link to the expected genetic gains over generations. We estimated the *h*^2^ values for all traits at each postharvest time point (Table 1). Moderate *h*^2^ values were recorded for firmness across all time points. For example, we observed an *h*^2^ of 0.54 for firmness at 1D, supporting previous results reported for blueberries (Cellon *et al*. 2018; Cappai *et al*. 2020). Low-to-moderate heritability values were also observed for Δfirmness, increasing from 0.29 at 1W to 0.45 at 7W. Notably, *h*^2^ of 0.7 was reported for Δfirmness after 4 weeks of storage at 4°C using a biparental population (Cappai *et al*. 2020). Moderate to high *h*^2^ has also been reported for firmness or postharvest softening in other fruit crops like apple, pear, sweet cherry, and strawberry (Shaw *et al*. 1987; Iwanami *et al*. 2008; Piaskowski *et al*. 2018; Kumar *et al*. 2019).

Altogether, our results suggest that firmness and its variation during postharvest storage are genetically controlled. The findings present promising opportunities for the breeding program to develop genotypes that not only exhibit high firmness at harvest but also demonstrate a slower softening rate during postharvest handling, leading to a prolonged shelf life. However, it is important to note that firmness might be influenced by strong genetic-by-postharvest-time interactions. This fact is supported by significant LRT results when complete and reduced models were compared for the importance of the genetic-by-time interaction (Table 1). Additionally, we used a post hoc test to quantify the proportion of ranking change across time points and noticed that 25% of the population exhibited crossover interactions in their breeding values between 1D and 7W (see *Ψ*_1d–7w_ in Table 1 and Supplementary Fig. 5). Consequently, it is crucial to continue evaluating and selecting for these traits at multiple postharvest time points in order to achieve high selection accuracy and, ultimately, a desirable shelf life.

A different phenomenon was observed in the TTA, which showed a decreasing *h*^2^ across the postharvest storage period, dropping from 0.54 at 1D to 0.48 at 7W. As shown in Fig. 2, the TTA is a trait that is not stable during postharvest storage, tending to decrease over time. However, these reductions were found to be lowly heritable (*h*^2^ = 0.10 at 7W), suggesting that variations in fruit acidity are mostly influenced by nongenetic factors. This is also supported by the low proportion of crossover events (*Ψ*_1d–7w_ = 0.01). Cultivars that decrease their fruit's acidity during postharvest storage would be a good option for shipping to distant markets that demand low-acidity berries. However, the low heritability value suggests that the genetic architecture of this trait is complex, making genetic improvement challenging. Nevertheless, some individuals with a high rate of TTA decrease during postharvest were identified and can be used for further study. Alternatively, selecting genotypes with a low initial TTA, which has higher heritability, would be more impactful in reducing the TTA in the short term.

Bloom is another postharvest trait of high breeding interest because it is considered an index of freshness at the retailer and wholesaler levels. We observed low *h*^2^ across the postharvest time points. We hypothesize that this is due to the trait being visually scored, which introduces a high level of subjectivity and error (Krzanowski 1983). For future analyses, we plan to use computer vision combined with machine learning methods to improve the phenotyping accuracy of this trait.

### How are postharvest traits related?

**Fig. 3.**
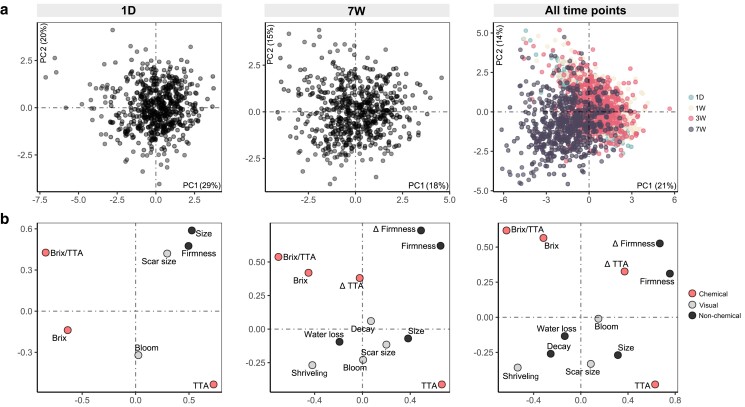
Principal component analysis plot showing the relationship of a) individuals and b) traits within a blueberry breeding population evaluated at 4 postharvest time points at 1°C. Principal component analysis plots were created using the FactoMineR package (Lê *et al*. 2008).

Using phenotypic data, principal component analyses were conducted to study the relationship between the traits and the individuals within and among the postharvest storage time points (Fig. 3a). The first 2 principal components explained 49% of the total phenotypic variance in the 1D treatment, decreasing to 33% in the 7W treatment. The high variability was observed among the genotypes at all time points without any noticeable clusters of individuals. When plotted together, a displacement of the 7W individuals was observed compared to the other 3 treatments. The 7W group clustered closer to senescence-related traits like water loss, decay, and shriveling and moved farther from traits like firmness and Δfirmness.

While most breeders have focused on breeding values to make selections, the phenotypic correlations between traits can express the extent to which 2 traits are related and might be effectively used for indirect selection when the targeted trait is difficult or expensive to phenotype (Fig. 3b). By initially focusing on firmness, we observed a low-to-moderate correlation between fruit size and firmness, especially at the 1D treatment, which goes against the general perception that cultivars with larger berries tend to be softer. A similar trend, but reported using genetic correlations, was described by Cellon *et al*. (2018). Regarding the variation in firmness during the postharvest, Δfirmness was not highly correlated with water loss (*r* = −0.05). This result contrasts with other studies reported in the literature, in which water loss is suggested as the major cause of fruit softening during postharvest storage (Paniagua *et al*. 2013). As an important observation, the lack of correlation between Δfirmness and water loss does not mean that controlling water loss is not critical during blueberry postharvest. Reports have shown that, within a genotype, water loss decreases the firmness of blueberries (Paniagua *et al*. 2013; Rivera *et al*. 2021). What this result suggests is that water loss is not an effective parameter for selecting genotypes with low softening rates in our population. We reinforce that further studies about cell wall degradation or reactive oxygen species metabolism are necessary to better understand the physiological factors behind the genetic variation in firmness during postharvest storage.

Other key traits of blueberries are sugar and acid concentrations. A recent study reported that such chemicals could explain a large portion of consumer preferences (Colantonio *et al*. 2022). In this study, we observed no phenotypic correlation between chemical traits and firmness or Δfirmness. Remarkably, this suggests that long-shelf life cultivars could be bred without sacrificing the organoleptic quality that consumers demand. A correlogram, including all the phenotypic correlations of the traits measured in this study, can be found in Supplementary Fig. 4.

### Fruit quality can be predicted along postharvest storage using genome-based models

**Fig. 4.**
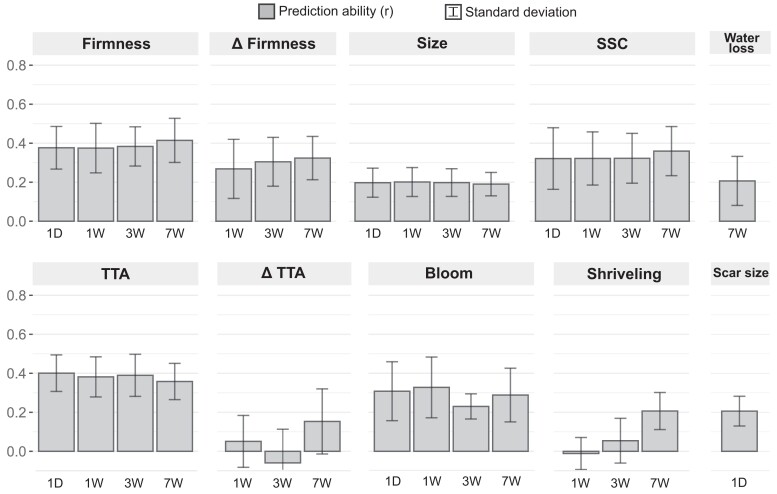
PA of the longitudinal genomic prediction models (GBLUP) for fruit quality traits across 4 postharvest storage time points at 1°C. Prediction abilities are represented by the mean Pearson correlation of the GEBVs and the phenotypic values using a 10-fold cross-validation in a CV1 scheme for each trait and time point.

The PA was estimated via genomic prediction within each postharvest time point using a 10-fold cross-validation in a CV1 scheme (Fig. 4). Similar to the narrow-sense heritability (Table 1), the PA did not decrease during postharvest for all traits. This suggests that genomic prediction could be effectively implemented for assisting selection, including those complex traits that exhibited higher crossover interactions during postharvest storage, such as firmness and Δfirmness.

For firmness, we noticed moderate PAs within all its postharvest time points, starting from 0.38 at 1D and increasing to 0.41 at 7W. A higher PA (0.45) for firmness at harvest was reported by Ferrão *et al*. (2021), but by using a larger blueberry breeding population phenotyped in multiple years. Importantly, the variation in firmness during storage can also be predicted with a low-to-moderate accuracy, achieving PAs of 0.30 and 0.32 at 3W and 7W, respectively. The low phenotypic correlation between initial firmness and Δfirmness suggests that both traits may be controlled by different genomic loci in blueberry (Supplementary Fig. 4). Hence, designing future crosses with the aim of accumulating favorable alleles for both traits would be an ideal approach for developing long-shelf life genotypes, particularly since genomics can aid in the selection process.

Among all the traits, the TTA recorded the highest PA at harvest, initiating at 0.40 and declining to 0.36 by the 7W postharvest time point. Lower values were presented by de Bem Oliveira *et al*. (2019) when predicting pH (0.28) in a population of 1,800 individuals, suggesting that the TTA could be a better-targeted trait in predictive breeding. Genomic information could not accurately predict ΔTTA for the 1W and 3W traits, an expected outcome given its limited variation and low heritability during those intervals. Nonetheless, by the 7W time point, the model successfully predicted ΔTTA with a PA of 0.15. Although the value is relatively low, it holds potential to streamline the difficult process of breeding blueberries that decline fruit acidity during postharvest storage.

**Fig. 5.**
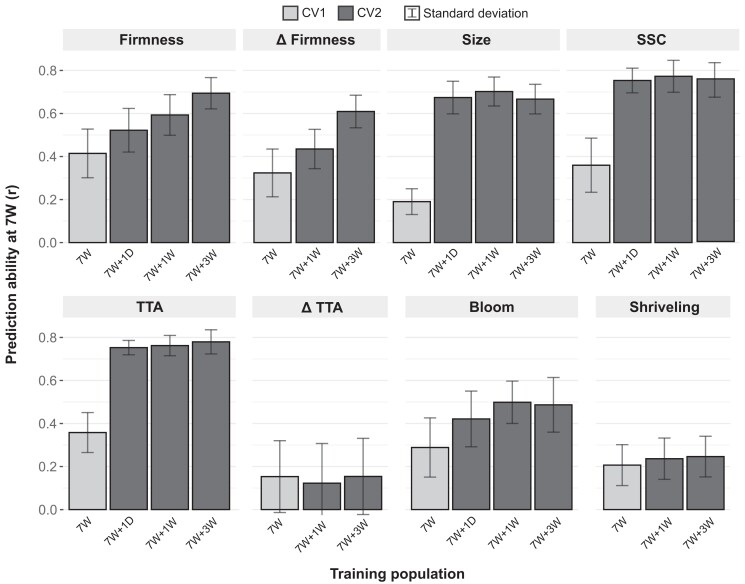
PA of the longitudinal genomic prediction models (GBLUP) for fruit quality traits measured after 7W of postharvest storage at 1°C. The models were trained using a CV2 scheme incorporating phenotypic data from previous time points. The results from the CV1 approach at 7W were also included for reference. Prediction abilities are represented by the mean Pearson correlation between the GEBVs and the phenotypic values following a 10-fold cross-validation approach.

One of the key advantages of using longitudinal mixed models is their ability to account for relatedness between factor levels. Classical methods, which assume independence between residuals, can lead to erroneous interpretations of genetic parameters in longitudinal experiments (Powers and Kozak 2019). Although our findings showed no significant differences in the PA when comparing longitudinal models to univariate models in a CV1 approach (Supplementary Fig. 6), the true benefit of longitudinal models became evident in a CV2 approach. Longitudinal models allow for the integration of data from all postharvest time points within a single model, enabling training with data collected at various intervals. In our breeding program, we annually collect phenotypic data on the fruit quality at harvest from our advanced selections. This practice prompted several questions: Can these harvest data enhance our postharvest predictions? When is the optimal time to measure these data—immediately after harvest (1D) or after a longer period (1W)? To address these questions, we implemented the same longitudinal model using a CV2 approach, considering the known phenotype of the predicted genotypes at an earlier time point (Fig. 5).

All models significantly increased their PA in the CV2 approach, except for ΔTTA and shriveling. This can be attributed to the low phenotypic variation of these traits at earlier time points. The prediction models for the fruit size, SSC, and TTA showed the highest increase in PA and demonstrated a consistent performance, regardless of whether they were trained with data from 1D, 1W, or 3W. This was expected due to the low variation of these traits during postharvest storage, resulting in high genetic correlations between their postharvest time points (Supplementary Table 4).

Including phenotypic data from earlier time points in the models’ training significantly increased the PA of firmness predictions. PAs increased from 0.41, when no previous phenotypic data were included, to 0.52, 0.59, and 0.69 when firmness data from 1D, 1W, or 7W was integrated, respectively. Similar results were observed for Δfirmness, where PA increased from 0.32 to 0.43 and 0.61 when the data from 1D or 3W were included. This improvement can be attributed to a decrease in the proportion of crossover interactions and higher genetic correlations between time points that are closer in time to each other (Supplementary Tables 3 and 4). These findings demonstrate that integrating phenotypic data obtained at harvest or after short postharvest storage periods into longitudinal genomic prediction models can be a valuable strategy to improve predictions of firmness and Δfirmness after long postharvest periods, which are critical traits for defining shelf life. To our knowledge, no longitudinal genomic prediction studies have been reported in fresh fruit postharvest research. However, other studies applying multivariate prediction models have demonstrated that integrating information from different factor levels, such as traits or environments, can significantly improve PAs for specific traits (Nsibi *et al*. 2020; Jung *et al*. 2022; Li *et al*. 2023).

### Breeding for increasing postharvest quality

Breeding for postharvest fruit quality in blueberries presents significant logistical and financial challenges. The intensity of these challenges escalates with an increase in the number of measured traits and time points during postharvest storage, limiting the number of genotypes that can undergo phenotyping. Our study identified firmness and Δfirmness as the key traits varying during postharvest storage. The high genetic control over these variations suggests that the targeted selection of these 2 traits can effectively enhance postharvest quality through breeding, especially considering that they were not correlated.

While genomic selection shows promise for predicting the postharvest quality, it cannot entirely replace the phenotyping of these key traits. The primary reasons are the low-to-moderate PAs and the pronounced genotype-by-time crossover interactions. Moreover, we noticed that PAs tend to decrease when applied to actual validation populations, as opposed to the more optimistic estimates obtained via 10-fold cross-validation (de Bem Oliveira *et al*. 2019; Ferrão *et al*. 2021). In this context, we envision the use of molecular breeding for prescreening and narrowing down high-performing individuals from expansive breeding populations prior to postharvest trials. This strategy would minimize the number of underperforming individuals subjected to phenotyping, thereby enhancing the efficiency of identifying top postharvest performers.

Longitudinal prediction models showed promising results for increasing the PA of firmness when phenotypic data at harvest are integrated into the models. This strategy could be highly beneficial for predicting the postharvest firmness of advanced selections that our breeding program has historically phenotyped at a single time point. Notably, we observed that including phenotypic data collected 1W after harvest, rather than data collected at harvest (1D), resulted in an increase of 0.07 in the PA and a decrease of 0.06 in crossover interactions of the breeding values. These findings suggest that shifting the breeding program's phenotyping protocol from “at harvest” to 1W postharvest could not only improve our predictions but also be more effective in selecting genotypes that retain high quality by the time they reach the consumer.

We found that firmness exhibited the highest genotype-by-time interaction across postharvest time points. Some genotypes performed better at specific time points. For example, the genotype “UF 17–166” showed moderate firmness at harvest but increased its firmness to become the best performer at 7W postharvest (Fig. 2a). In contrast, other genotypes, such as the recent UF cultivar ‘Colossus’, performed well and remained stable across all postharvest time points, although ‘Colossus’ did not perform as well as “UF 17–166” at the 7W time point. Additionally, genotypes like “UF 17–012” had the highest firmness until the 3W time point but became unmarketably soft by 7W.

This raises the question of which genotypes should be selected for future cultivar development. We believe there is no single correct answer as it depends on the objective of the breeding program and its target population of environments. For instance, if a breeding program is established in a location where growers mainly export their production to distant markets, selecting the genotype that outperforms after 7W of cold storage might be the optimal decision. On the other hand, for growers that supply both local and distant markets, a more stable genotype might be more appealing. Overall, our findings suggest that considering the genotype-by-time interaction effect is essential for optimizing the development of cultivars with a good postharvest quality.

## Conclusion

Blueberry breeding programs have been framed into recurrent selection strategies with 2 central goals: (i) identifying the best-performing genotypes for commercial release and (ii) detecting materials that can be used as parents in future crosses. To make this process more efficient, modern breeding programs have relied on understanding the genetic architecture of complex traits to assist future decisions. Aiming to provide new insights into the genetic bases of blueberry traits under postharvest conditions, the main contributions of this project are as follows: (i) after estimating genetic parameters using large breeding populations of SHB, most of the traits presented high heritability, suggesting that phenotypic variation is highly genetically controlled and therefore subject to fast genetic gains; (ii) large phenotypic variation and a high rate of crossover genotype-by-time interaction events were observed for firmness across the storage time points, a fact that indicates that phenotypes measured at harvest might not be a good indicator of the postharvest performance of a genotype; (iii) low correlations were found between the genetic effects of the chemical and textural attributes, suggesting that fruit quality and postharvest traits can be genetically improved simultaneously; (iv) the genomic prediction accuracy of key traits was consistent along the postharvest time points, suggesting that the postharvest quality can also be improved through predictive breeding; and (v) longitudinal genomic prediction models can leverage the PA of traits with strong genetic-by-time interactions when phenotypic data from previous time points are integrated into the models.

## Supplementary Material

jkae163_Supplementary_Data

## Data Availability

Supplementary File 1 contains a description of the phenotypic data file. The phenotypic data used in the analysis are available in Supplementary File 2. Supplementary File 3 contains the genotypic data matrix used in the analyses. Genotype names were recoded for intellectual property purposes. Supplemental material available at G3 online.
