# Supplementary material for: Understanding the genetic basis of blueberry postharvest traits to define better breeding strategies
Source: G3 (Bethesda). 2024 Jul 25;14(9):jkae163. doi: 10.1093/g3journal/jkae163 (PMC11373639; doi:10.1093/g3journal/jkae163)
Supplement: jkae163_Supplementary_Data [file jkae163_supplementary_data.zip › Figure_S1_G3-2024-405222.docx]

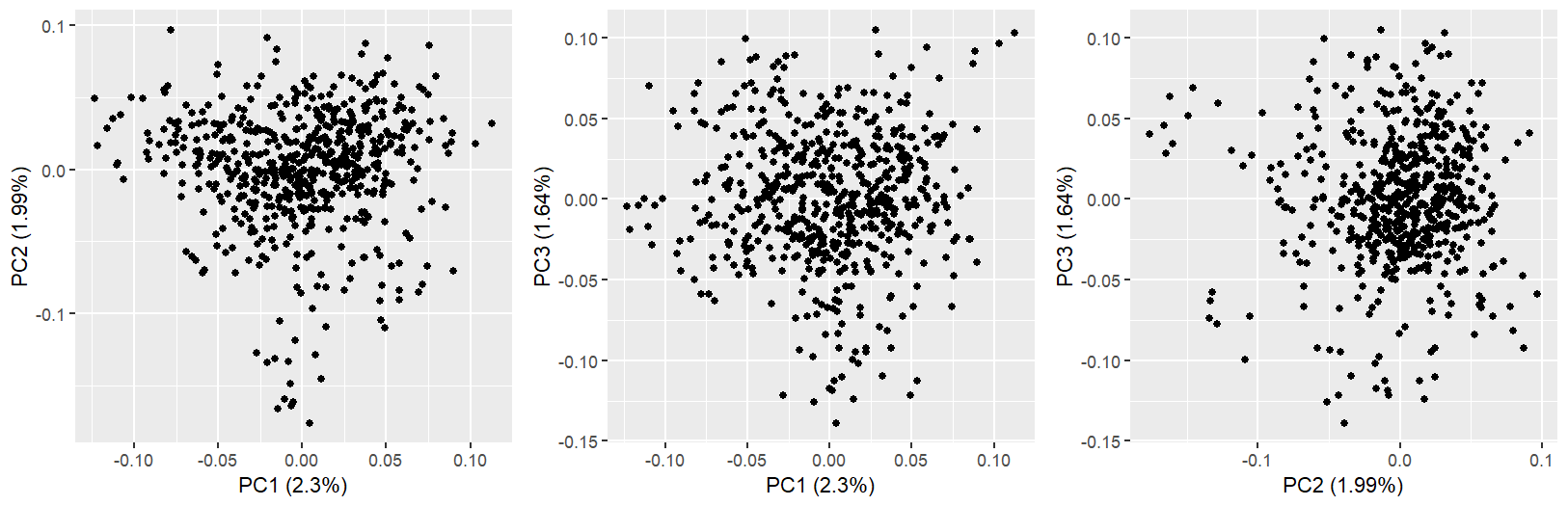


**Figure S1.** Principal component analysis of the molecular marker data. Percentage inside the brackets indicates the proportion of the variance explained by each principal component.
