# Supplementary material for: Understanding the genetic basis of blueberry postharvest traits to define better breeding strategies
Source: G3 (Bethesda). 2024 Jul 25;14(9):jkae163. doi: 10.1093/g3journal/jkae163 (PMC11373639; doi:10.1093/g3journal/jkae163)
Supplement: jkae163_Supplementary_Data [file jkae163_supplementary_data.zip › Figure_S2_G3-2024-405222.docx]

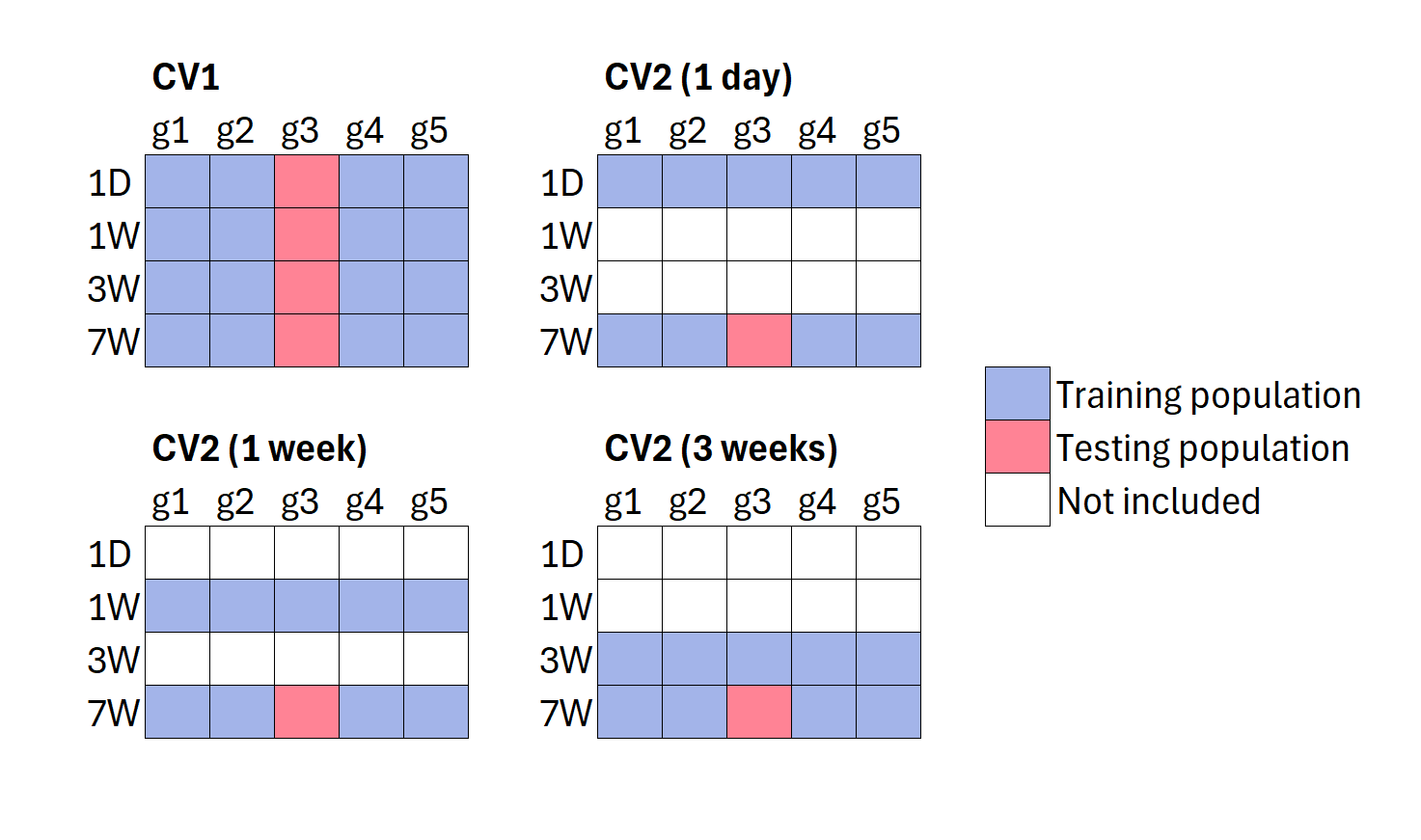


**Figure S2.** Graphical representation of the cross-validation schemes tested in the genomic prediction analysis. A traditional CV1 approach, which predicted genotypes without phenotypic data at any time point, and three CV2 scenarios where genotypes at 7 weeks postharvest were predicted, knowing their phenotype at different earlier time points (1 day, 1 week, or 3 weeks postharvest).
