# Supplementary material for: Understanding the genetic basis of blueberry postharvest traits to define better breeding strategies
Source: G3 (Bethesda). 2024 Jul 25;14(9):jkae163. doi: 10.1093/g3journal/jkae163 (PMC11373639; doi:10.1093/g3journal/jkae163)
Supplement: jkae163_Supplementary_Data [file jkae163_supplementary_data.zip › Figure_S3_G3-2024-405222.docx]

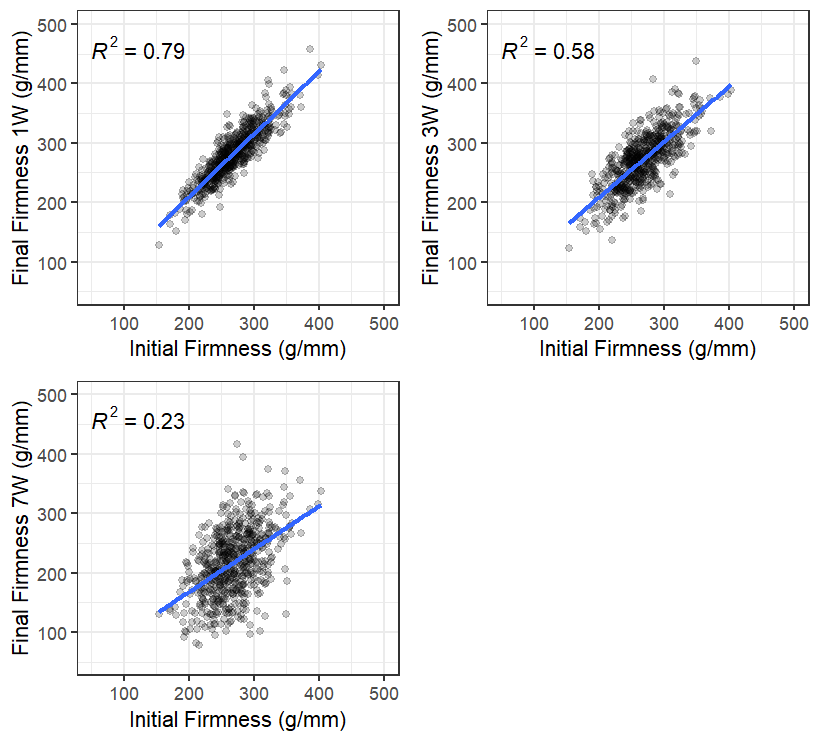


**Figure S3.** Linear regression between initial and final blueberry firmness after one week (1W), three weeks (3W), and seven weeks (7W) of postharvest storage at 1°C. Each point represents a single genotype. Initial firmness was measured after 1 day of postharvest storage. All regressions were significant (p<0.001).
