# Supplementary material for: Understanding the genetic basis of blueberry postharvest traits to define better breeding strategies
Source: G3 (Bethesda). 2024 Jul 25;14(9):jkae163. doi: 10.1093/g3journal/jkae163 (PMC11373639; doi:10.1093/g3journal/jkae163)
Supplement: jkae163_Supplementary_Data [file jkae163_supplementary_data.zip › Figure_S4_G3-2024-405222.docx]

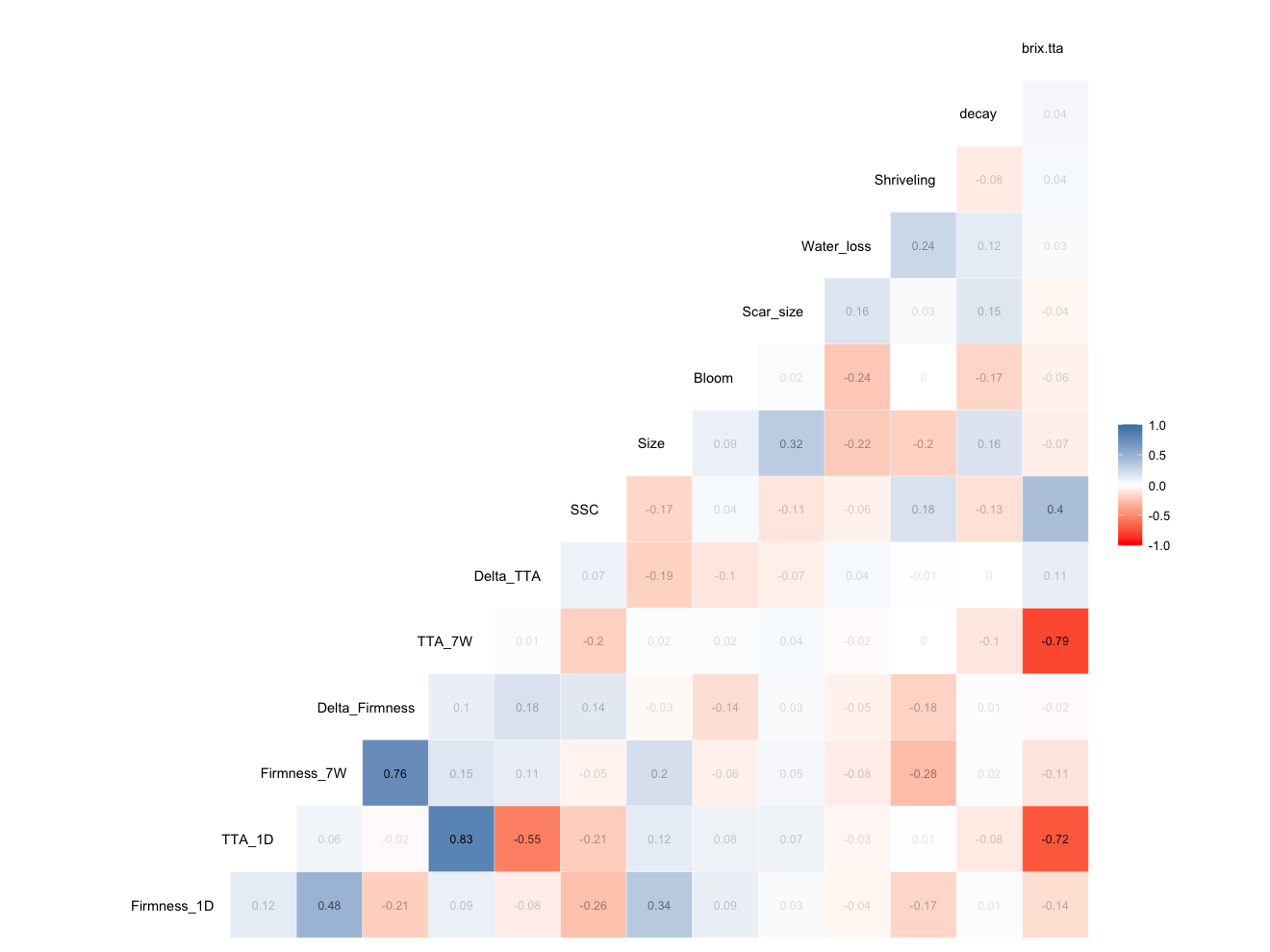


**Figure S4.** Correlations of phenotypic values for traits at 7 weeks of postharvest storage at 1°C, including fruit firmness and total titratable acidity (TTA) at 1 day of storage. Delta_Firmness and Delta_TTA represent the variation of those two traits during postharvest, where a positive value indicates an increase in the trait magnitude over time, and a negative value indicates a decrease.
