# Supplementary material for: Understanding the genetic basis of blueberry postharvest traits to define better breeding strategies
Source: G3 (Bethesda). 2024 Jul 25;14(9):jkae163. doi: 10.1093/g3journal/jkae163 (PMC11373639; doi:10.1093/g3journal/jkae163)
Supplement: jkae163_Supplementary_Data [file jkae163_supplementary_data.zip › Figure_S6_G3-2024-405222.docx]

Prediction accuracy (r)


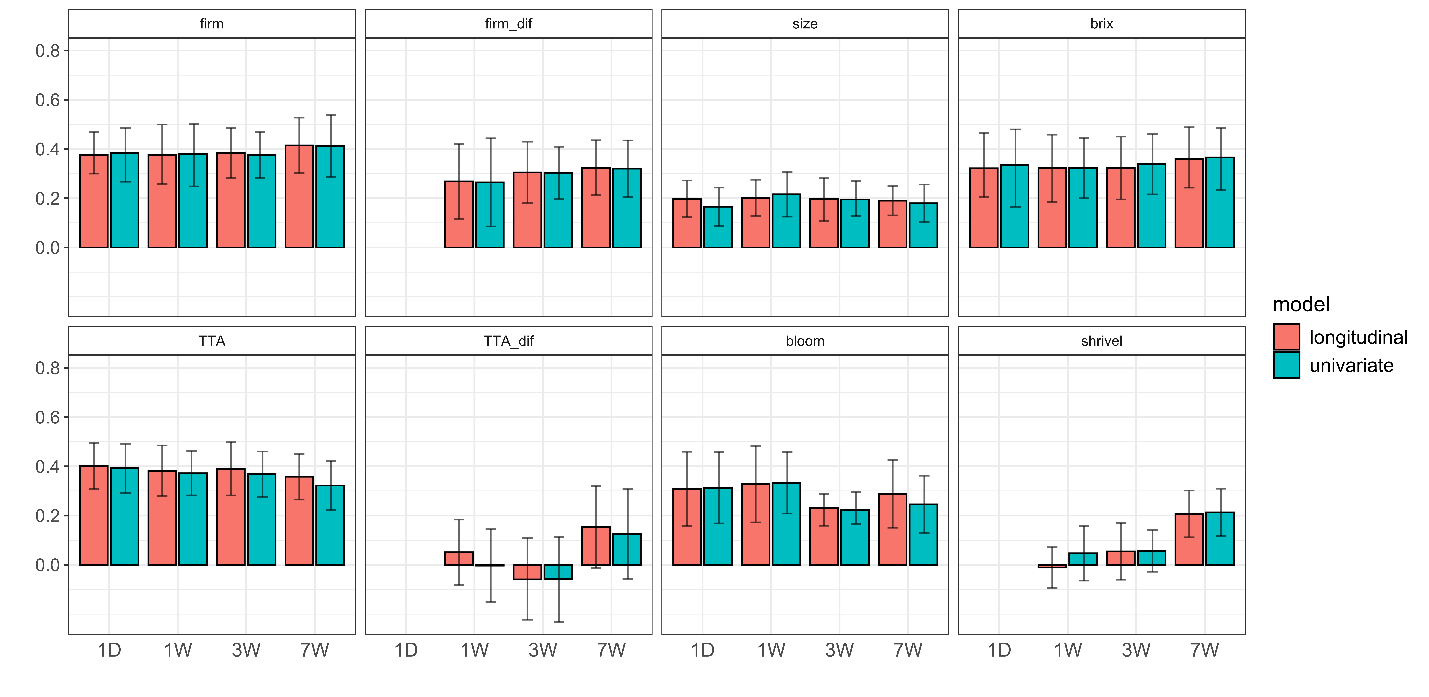


**Figure S6.** Mean prediction accuracy (r) of univariate and longitudinal genomic prediction models. Results are based on a 10-fold CV1 scheme. Whiskers represent the standard deviation of the mean.
