# Supplementary material for: Understanding the genetic basis of blueberry postharvest traits to define better breeding strategies
Source: G3 (Bethesda). 2024 Jul 25;14(9):jkae163. doi: 10.1093/g3journal/jkae163 (PMC11373639; doi:10.1093/g3journal/jkae163)
Supplement: jkae163_Supplementary_Data [file jkae163_supplementary_data.zip › Supplemental_Material_Legends_G3-2024-405222.docx]

**Supplemental materials**

1. **Figures**


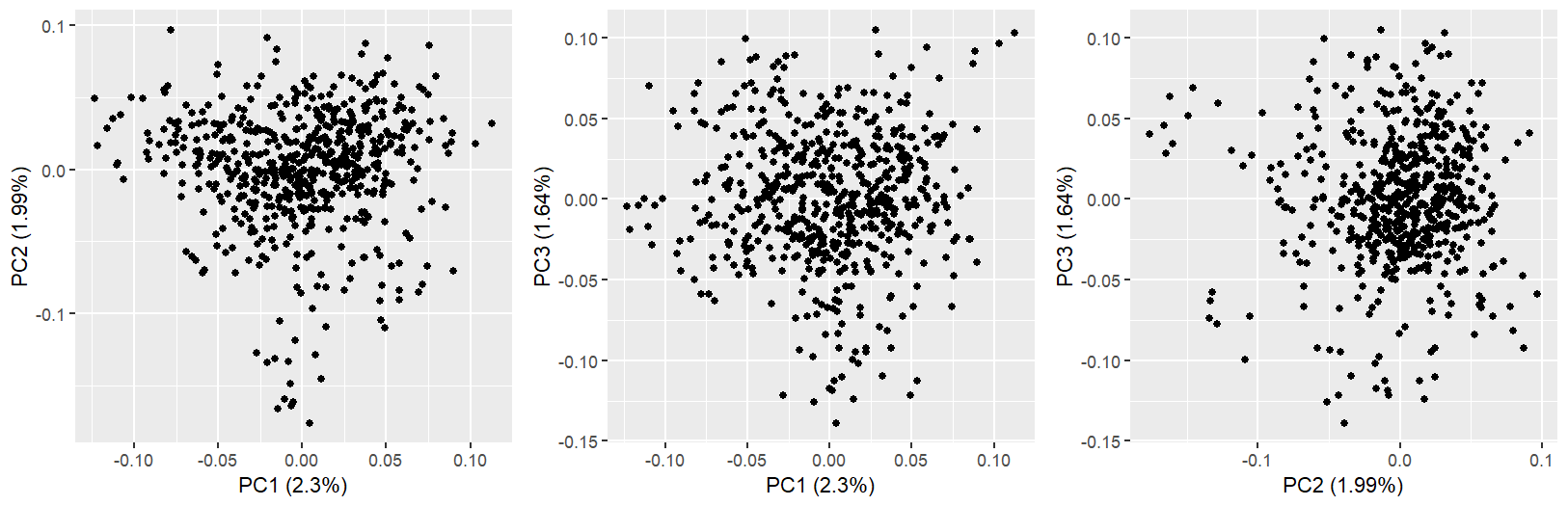


**Figure S1.** Principal component analysis of the molecular marker data. Percentage inside the brackets indicates the proportion of the variance explained by each principal component.


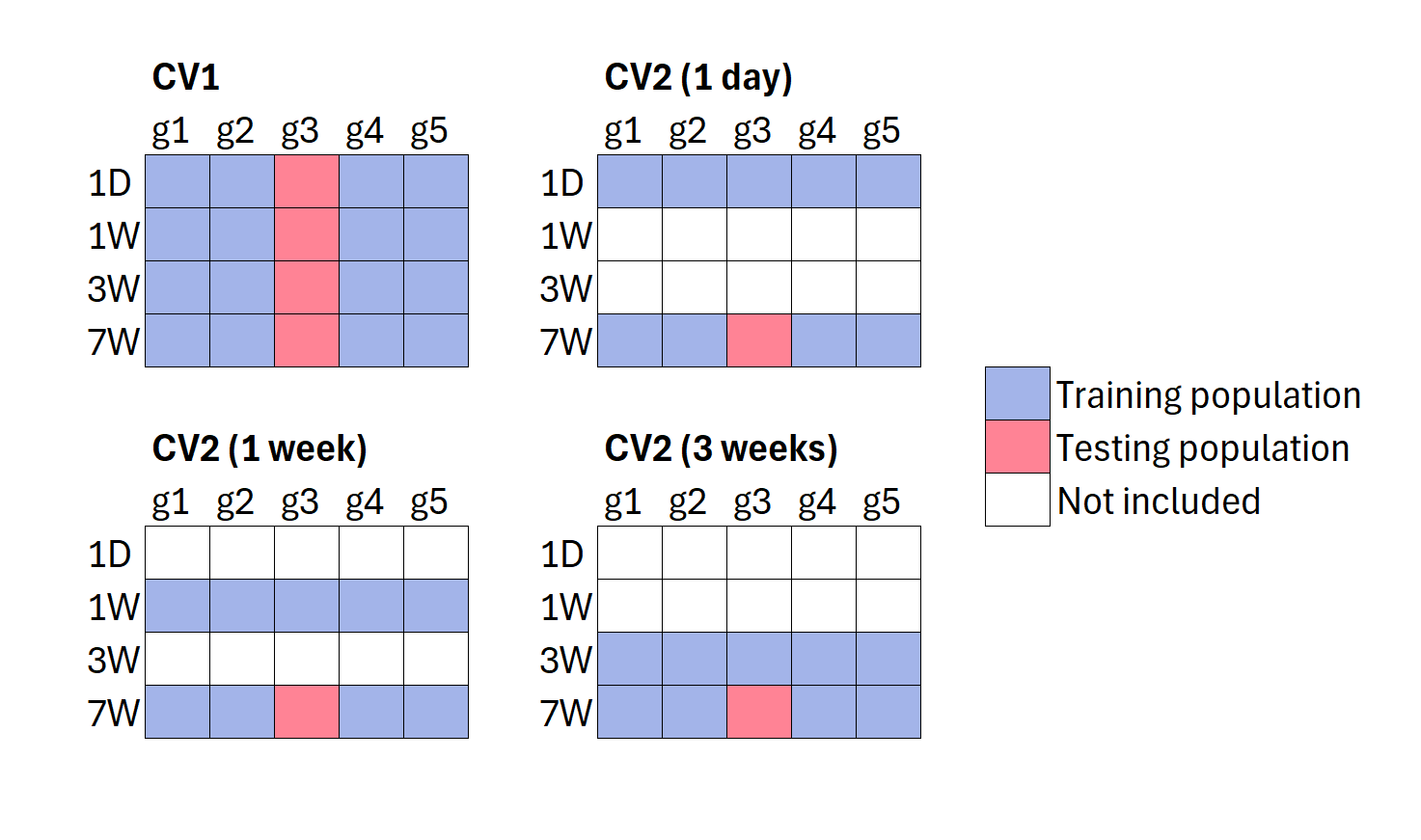


**Figure S2.** Graphical representation of the cross-validation schemes tested in the genomic prediction analysis. A traditional CV1 approach, which predicted genotypes without phenotypic data at any time point, and three CV2 scenarios where genotypes at 7 weeks postharvest were predicted, knowing their phenotype at different earlier time points (1 day, 1 week, or 3 weeks postharvest).


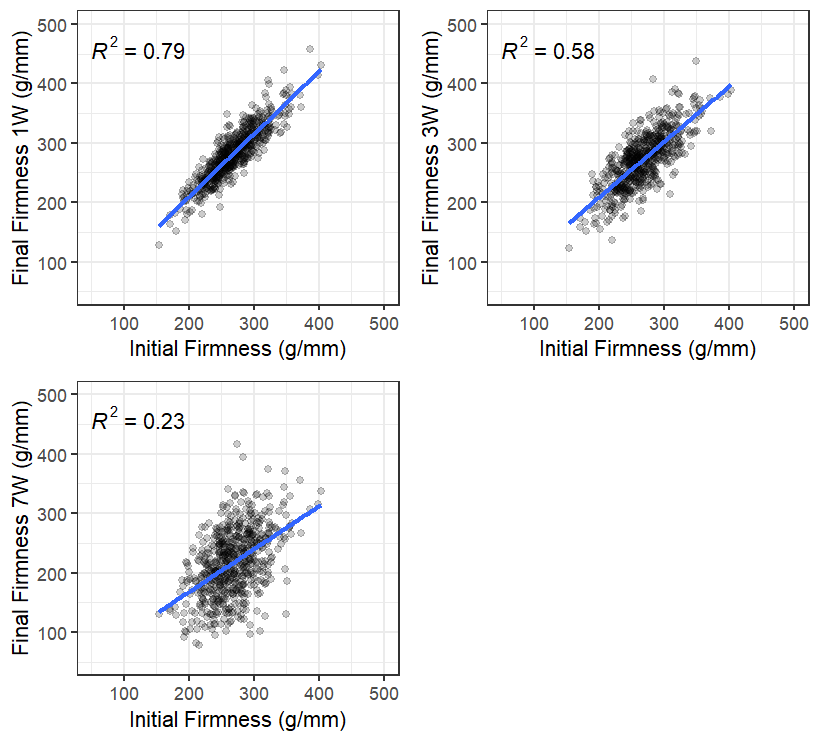


**Figure S3.** Linear regression between initial and final blueberry firmness after one week (1W), three weeks (3W), and seven weeks (7W) of postharvest storage at 1°C. Each point represents a single genotype. Initial firmness was measured after 1 day of postharvest storage. All regressions were significant (p<0.001).


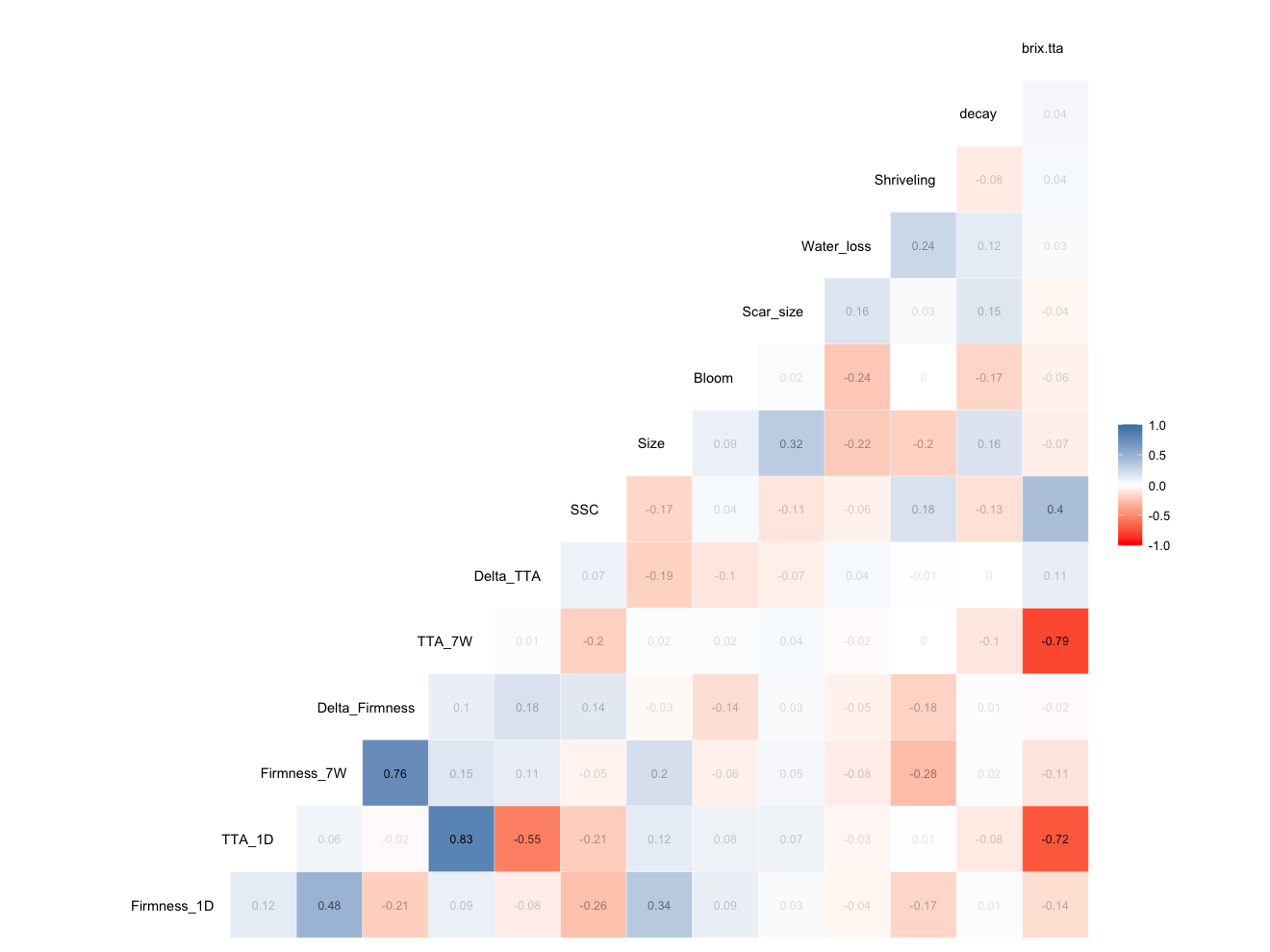


**Figure S4.** Correlations of phenotypic values for traits at 7 weeks of postharvest storage at 1°C, including fruit firmness and total titratable acidity (TTA) at 1 day of storage. Delta_Firmness and Delta_TTA represent the variation of those two traits during postharvest, where a positive value indicates an increase in the trait magnitude over time, and a negative value indicates a decrease.


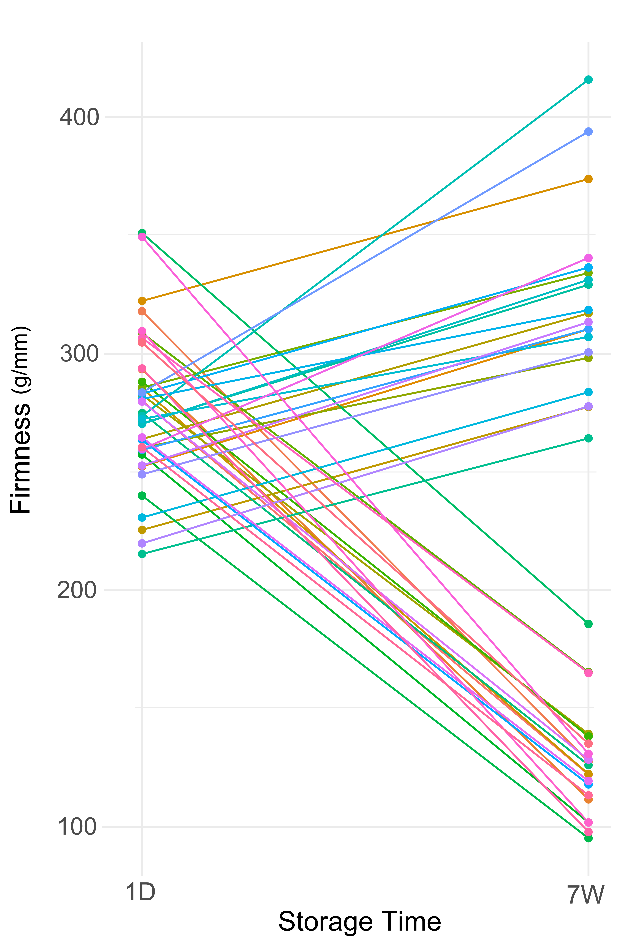


**Figure S5.** Representation of crossover-type genotype-by-time interaction by displaying the experiment's top-25 firming genotypes and top-25 softening genotypes.

Prediction accuracy (r)


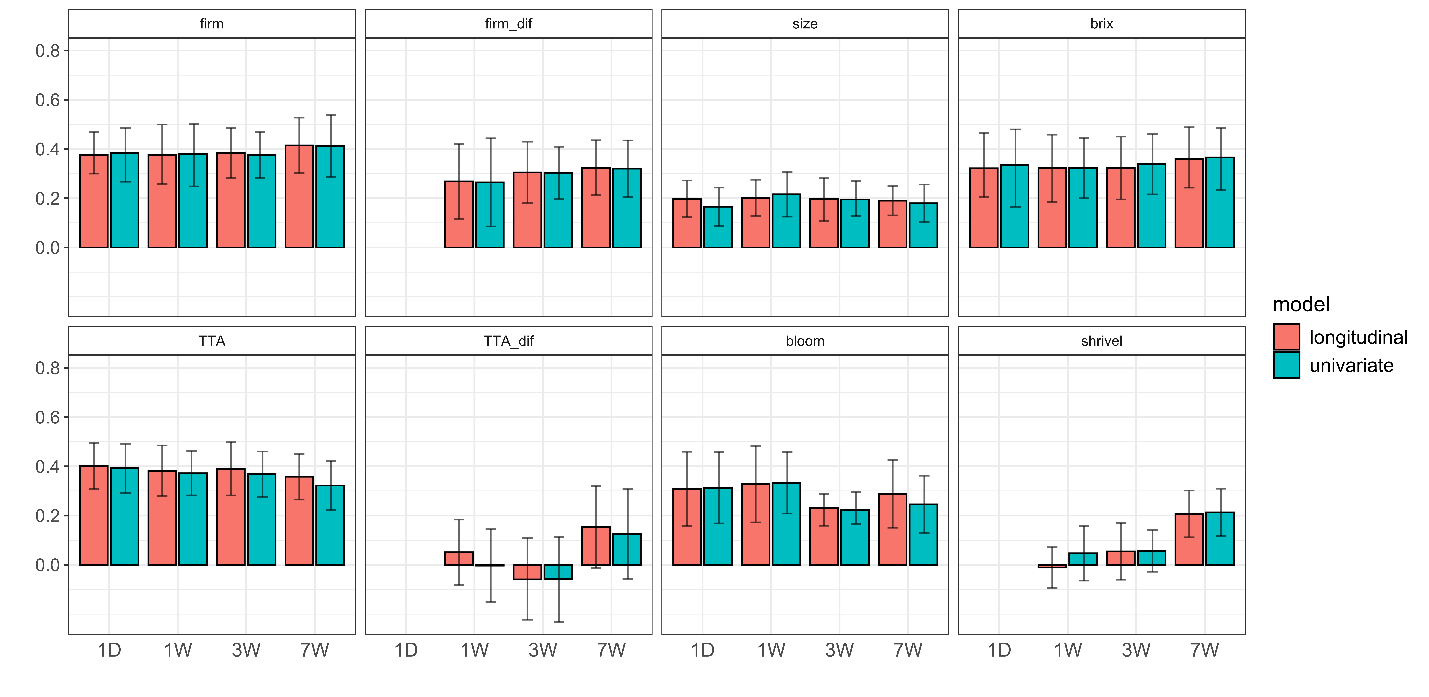


**Figure S6.** Mean prediction accuracy (r) of univariate and longitudinal genomic prediction models. Results are based on a 10-fold CV1 scheme. Whiskers represent the standard deviation of the mean.

1. **Tables**

**Table S1.** Bayesian Information Criterion (BIC) values for model selection using two different (co)variance structures: "corgh" represents an unstructured correlation with heterogeneous variance, and "diag" represents independent effects with heterogeneous variances. The (co)variances were modeled in both random effects and residuals. Additionally, the correlation between the BLUPs computed by both models is shown.

| Trait | BIC_corgh_ | BIC_diag_ | BLUPs correlation |
| --- | --- | --- | --- |
| Firmness (g/mm) | 19422 | 21352 | 0.97 |
| TTA (%) | -8313 | -5687 | 0.96 |
| SSC (brix) | 306 | 3528 | 0.97 |
| Size (mm) | 744 | 3710 | 0.97 |
| Bloom (score) | -598 | -135 | 0.90 |
| ΔFirmness (g/mm) | 14312 | 14842 | 0.98 |
| ΔTTA (%) | -6665 | -6181 | 0.85 |
| Shriveling (score) | -2345 | -2282 | 0.98 |

**Table S2.** Genetic (σ_g_) and residual (σ_e_) variance components for each trait at four postharvest time points (1 day, 1 week, 3 weeks, and 7 weeks). Variance components were estimated using restricted maximum likelihood (REML) in a longitudinal linear mixed model. Traits not measured at specific time points are indicated with a dash (-).

| Trait | σ_g_ 1D | σ_g_ 1W | σ_g_ 3W | σ_g_ 7W | σ_e_ 1D | σ_e_ 1W | σ_e_ 3W | σ_e_ 7W |
| --- | --- | --- | --- | --- | --- | --- | --- | --- |
| Firmness (g/mm) | 713.7 | 924.2 | 1018.1 | 1788.2 | 616.6 | 944.2 | 1031.8 | 1344.9 |
| TTA (%) | 0.028 | 0.024 | 0.025 | 0.016 | 0.021 | 0.023 | 0.022 | 0.018 |
| SSC (brix) | 0.584 | 0.611 | 0.614 | 0.700 | 1.033 | 0.967 | 1.016 | 0.875 |
| Size (mm) | 0.714 | 0.782 | 0.666 | 0.584 | 1.006 | 1.057 | 1.054 | 1.016 |
| Bloom (score) | 0.087 | 0.093 | 0.091 | 0.115 | 0.210 | 0.257 | 0.260 | 0.309 |
| ΔFirmness (g/mm) | - | 121.5 | 324.5 | 1200.3 | - | 293.9 | 591.2 | 1460.0 |
| ΔTTA (%) | - | 0.000 | 0.000 | 0.002 | - | 0.010 | 0.014 | 0.015 |
| Shriveling (score) | - | 0.002 | 0.010 | 0.072 | - | 0.041 | 0.091 | 0.201 |
| Water loss (%) | - | - | - | 0.126 | - | - | - | 0.576 |
| Scar size (score) | 0.074 | - | - | - | 0.100 | - | - | - |

**Table S3.** Proportion of crossover-type genotype-by-time interactions of breeding values (Ψ) in the population between pairs of postharvest time points (1 day, 1 week, 3 weeks, and 7 weeks). Additionally, p-values obtained by the likelihood ratio test (LRT) are shown, where a significant value addresses the presence of genotype-by-time interaction in the experiment.

| Trait | Ψ_1D~7W_ | Ψ_1W~7W_ | Ψ_3W~7W_ | LRT  (p-value) |
| --- | --- | --- | --- | --- |
| Firmness (g/mm) | 0.25 | 0.19 | 0.14 | < 0.001 |
| TTA (%) | 0.03 | 0.03 | 0.04 | < 0.001 |
| SSC (brix) | 0.03 | 0.02 | 0.03 | 0.104 |
| Bloom (score) | 0.13 | 0.12 | 0.09 | 0.042 |
| Size (mm) | 0.07 | 0.07 | 0.05 | 0.003 |
| ΔFirmness (g/mm) | - | 0.20 | 0.16 | < 0.001 |
| ΔTTA (%) | - | 0.01 | 0.01 | 0.002 |
| Shriveling (score) | - | - | 0.21 | < 0.001 |
|  |  |  |  |  |

**Table S4.** Genetic correlations (ρ) estimated using restricted maximum likelihood (REML) for each pair of postharvest time points in the longitudinal models.

| Trait | ρ_1d1w_ | ρ_1d3w_ | ρ_1d7w_ | ρ_1w3w_ | ρ_1w7w_ | ρ_3w7w_ |
| --- | --- | --- | --- | --- | --- | --- |
| Firmness (g/mm) | 0.957 | 0.854 | 0.610 | 0.945 | 0.746 | 0.841 |
| TTA (%) | 0.999 | 0.998 | 0.984 | 0.999 | 0.999 | 0.968 |
| SSC (brix) | 0.998 | 0.998 | 0.985 | 0.999 | 0.996 | 0.979 |
| Size (mm) | 0.999 | 0.999 | 0.957 | 0.999 | 0.956 | 0.986 |
| Bloom (visual score) | 0.881 | 0.655 | 0.896 | 0.733 | 0.873 | 0.999 |
| ΔFirmness (g/mm) | - | - | - | 0.465 | 0.218 | 0.581 |
| ΔTTA (%) | - | - | - | 0.513 | 0.448 | 0.666 |
| Shriveling (score) | - | - | - | 0.249 | 0.142 | 0.217 |

**Statistical Models**

A longitudinal linear mixed model was fitted using all the postharvest time points and their interaction with the genotypic effect simultaneously. The model was fitted in the asreml-R package (Butler et al., 2018), as follows:

$\mathbf{y}\boldsymbol{=}\boldsymbol{X}_{\boldsymbol{1}}\boldsymbol{\beta+}\boldsymbol{X}_{\boldsymbol{2}}\boldsymbol{t+}\boldsymbol{X}_{\boldsymbol{3}}\boldsymbol{g}_{\boldsymbol{1}}\boldsymbol{+Z}\boldsymbol{g}_{\boldsymbol{2}}\boldsymbol{+e}$ ;

where $\mathbf{y}$ **(**$\boldsymbol{n\times1}$**)** is the vector of phenotypes at all time points, $\boldsymbol{\beta(}\left( \boldsymbol{n}_{\boldsymbol{year}}\boldsymbol{+1} \right)\boldsymbol{\times1)}$ is the vector of population mean at each time point plus the year of evaluation, and ***t*** $\left( \boldsymbol{n}_{\boldsymbol{time}}\boldsymbol{\times1} \right)$ is a vector of fixed effects of the postharvest period. We split the genotypic effect into two components, a similar approach to that reported by Pastina et al. (2012) and de C. Lara et al. (2019), $\boldsymbol{g}_{\boldsymbol{1}}\mathbf{(}\boldsymbol{n}\boldsymbol{\times1}\mathbf{)}$ is the vector of fixed effects of checks connecting the time points, $\boldsymbol{g}_{\boldsymbol{2}}$ $\boldsymbol{(n}_{\boldsymbol{time}}\boldsymbol{(}\boldsymbol{n}_{\boldsymbol{test}}\boldsymbol{+}\boldsymbol{n}_{\boldsymbol{check}}\boldsymbol{)\times}\boldsymbol{1}$**)** is the vector of random effects of test genotypes, and $\boldsymbol{e} \mathbf{(}\boldsymbol{n}\boldsymbol{\times}\boldsymbol{1}\mathbf{)}$ is the vector of random residual effects. The Gaussian distribution was assumed for the genetics ($\boldsymbol{g}_{\boldsymbol{2}}$) and residual effects ($\boldsymbol{e)}$, $\boldsymbol{g}_{\boldsymbol{2}}\boldsymbol{\sim MVN}\left( \boldsymbol{0,}\sum_{\boldsymbol{t}} \boldsymbol{\otimes G} \right)$ and $\boldsymbol{e\sim MVN}\left( \boldsymbol{0,}\sum_{\boldsymbol{e}} \boldsymbol{\otimes I} \right)$; where **G ((**$\boldsymbol{n}_{\boldsymbol{test}}\boldsymbol{+}\boldsymbol{n}_{\boldsymbol{checks}}$**)** $\boldsymbol{\times(}\boldsymbol{n}_{\boldsymbol{test}}\boldsymbol{+}\boldsymbol{n}_{\boldsymbol{checks}}$**))** is the additive relationship matrix calculated with molecular markers information and constructed using the Van Raden methodology assuming tetrasomic inheritance in the AGHmatrix R package (R. Amadeu et al., 2016). $\boldsymbol{I}$ $\left( \boldsymbol{n}_{\boldsymbol{year}}\boldsymbol{(}\boldsymbol{n}_{\boldsymbol{test}}\boldsymbol{+}\boldsymbol{n}_{\boldsymbol{checks}}\boldsymbol{)}\boldsymbol{\times}\boldsymbol{n}_{\boldsymbol{year}}\boldsymbol{(}\boldsymbol{n}_{\boldsymbol{test}}\boldsymbol{+}\boldsymbol{n}_{\boldsymbol{checks}}\boldsymbol{)} \right)$ is an identity matrix. $\sum_{\boldsymbol{t}} \left( \boldsymbol{n}_{\boldsymbol{time}}\boldsymbol{\times}\boldsymbol{n}_{\boldsymbol{time}} \right)$and $\sum_{\boldsymbol{e}} \left( \boldsymbol{n}_{\boldsymbol{time}}\boldsymbol{\times}\boldsymbol{n}_{\boldsymbol{time}} \right)$are unstructured variance-correlation structures for the postharvest time effect and the residuals, respectively. $\boldsymbol{X}_{\boldsymbol{1}}\boldsymbol{(}\boldsymbol{n\times}\left( \boldsymbol{n}_{\boldsymbol{year}}\boldsymbol{+1} \right)\boldsymbol{)}$**,** $\boldsymbol{X}_{\boldsymbol{2}}$**(**$\boldsymbol{n}\boldsymbol{\times}\boldsymbol{n}_{\boldsymbol{time}}$**)**, $\boldsymbol{X}_{\boldsymbol{3}}$ **(**$\boldsymbol{n}\boldsymbol{\times(}\boldsymbol{n}_{\boldsymbol{test}}\boldsymbol{+}\boldsymbol{n}_{\boldsymbol{checks}}$**))** and **Z (**$\boldsymbol{n}\boldsymbol{\times}\boldsymbol{n}_{\boldsymbol{time}}\boldsymbol{(}\boldsymbol{n}_{\boldsymbol{test}}\boldsymbol{+}\boldsymbol{n}_{\boldsymbol{checks}}$**))** are the incidence matrices. The dimensions are defined by the quantities: $\boldsymbol{n}=$number of observations across time points and years, $\boldsymbol{n}_{\boldsymbol{test}}=572$ is the test genotypes, $\boldsymbol{n}_{\boldsymbol{checks}}=16$ is the checks genotypes, $\boldsymbol{n}_{\boldsymbol{time}}=4$ is the number of time points, $\boldsymbol{n}_{\boldsymbol{year}}=3$ is the number of years. To assess the goodness-of-fit, a simpler model assuming no correlation between time points or residuals was fitted and compared to the more complex longitudinal model using the Bayesian Information Criterion (BIC) (Neath & Cavanaugh, 2012). The BIC was consistently lower for all traits when assuming an unstructured correlation in the model, indicating that the unstructured correlation model provides a better fit for the data (Table S1).
