# Supplementary material for: Understanding the genetic basis of blueberry postharvest traits to define better breeding strategies
Source: G3 (Bethesda). 2024 Jul 25;14(9):jkae163. doi: 10.1093/g3journal/jkae163 (PMC11373639; doi:10.1093/g3journal/jkae163)
Supplement: jkae163_Supplementary_Data [file jkae163_supplementary_data.zip › Table_S1_G3-2024-405222.docx]

**Table S1.** Bayesian Information Criterion (BIC) values for model selection using two different (co)variance structures: "corgh" represents an unstructured correlation with heterogeneous variance, and "diag" represents independent effects with heterogeneous variances. The (co)variances were modeled in both random effects and residuals. Additionally, the correlation between the BLUPs computed by both models is shown.

| Trait | BIC_corgh_ | BIC_diag_ | BLUPs correlation |
| --- | --- | --- | --- |
| Firmness (g/mm) | 19422 | 21352 | 0.97 |
| TTA (%) | -8313 | -5687 | 0.96 |
| SSC (brix) | 306 | 3528 | 0.97 |
| Size (mm) | 744 | 3710 | 0.97 |
| Bloom (score) | -598 | -135 | 0.90 |
| ΔFirmness (g/mm) | 14312 | 14842 | 0.98 |
| ΔTTA (%) | -6665 | -6181 | 0.85 |
| Shriveling (score) | -2345 | -2282 | 0.98 |
