# Supplementary material for: Understanding the genetic basis of blueberry postharvest traits to define better breeding strategies
Source: G3 (Bethesda). 2024 Jul 25;14(9):jkae163. doi: 10.1093/g3journal/jkae163 (PMC11373639; doi:10.1093/g3journal/jkae163)
Supplement: jkae163_Supplementary_Data [file jkae163_supplementary_data.zip › Table_S2_G3-2024-405222.docx]

**Table S2.** Genetic (σ_g_) and residual (σ_e_) variance components for each trait at four postharvest time points (1 day, 1 week, 3 weeks, and 7 weeks). Variance components were estimated using restricted maximum likelihood (REML) in a longitudinal linear mixed model. Traits not measured at specific time points are indicated with a dash (-).

| Trait | σ_g_ 1D | σ_g_ 1W | σ_g_ 3W | σ_g_ 7W | σ_e_ 1D | σ_e_ 1W | σ_e_ 3W | σ_e_ 7W |
| --- | --- | --- | --- | --- | --- | --- | --- | --- |
| Firmness (g/mm) | 713.7 | 924.2 | 1018.1 | 1788.2 | 616.6 | 944.2 | 1031.8 | 1344.9 |
| TTA (%) | 0.028 | 0.024 | 0.025 | 0.016 | 0.021 | 0.023 | 0.022 | 0.018 |
| SSC (brix) | 0.584 | 0.611 | 0.614 | 0.700 | 1.033 | 0.967 | 1.016 | 0.875 |
| Size (mm) | 0.714 | 0.782 | 0.666 | 0.584 | 1.006 | 1.057 | 1.054 | 1.016 |
| Bloom (score) | 0.087 | 0.093 | 0.091 | 0.115 | 0.210 | 0.257 | 0.260 | 0.309 |
| ΔFirmness (g/mm) | - | 121.5 | 324.5 | 1200.3 | - | 293.9 | 591.2 | 1460.0 |
| ΔTTA (%) | - | 0.000 | 0.000 | 0.002 | - | 0.010 | 0.014 | 0.015 |
| Shriveling (score) | - | 0.002 | 0.010 | 0.072 | - | 0.041 | 0.091 | 0.201 |
| Water loss (%) | - | - | - | 0.126 | - | - | - | 0.576 |
| Scar size (score) | 0.074 | - | - | - | 0.100 | - | - | - |
