# Supplementary material for: Understanding the genetic basis of blueberry postharvest traits to define better breeding strategies
Source: G3 (Bethesda). 2024 Jul 25;14(9):jkae163. doi: 10.1093/g3journal/jkae163 (PMC11373639; doi:10.1093/g3journal/jkae163)
Supplement: jkae163_Supplementary_Data [file jkae163_supplementary_data.zip › Table_S3_G3-2024-405222.docx]

**Table S3.** Proportion of crossover-type genotype-by-time interactions of breeding values (Ψ) in the population between pairs of postharvest time points (1 day, 1 week, 3 weeks, and 7 weeks). Additionally, p-values obtained by the likelihood ratio test (LRT) are shown, where a significant value addresses the presence of genotype-by-time interaction in the experiment.

| Trait | Ψ_1D~7W_ | Ψ_1W~7W_ | Ψ_3W~7W_ | LRT  (p-value) |
| --- | --- | --- | --- | --- |
| Firmness (g/mm) | 0.25 | 0.19 | 0.14 | < 0.001 |
| TTA (%) | 0.03 | 0.03 | 0.04 | < 0.001 |
| SSC (brix) | 0.03 | 0.02 | 0.03 | 0.104 |
| Bloom (score) | 0.13 | 0.12 | 0.09 | 0.042 |
| Size (mm) | 0.07 | 0.07 | 0.05 | 0.003 |
| ΔFirmness (g/mm) | - | 0.20 | 0.16 | < 0.001 |
| ΔTTA (%) | - | 0.01 | 0.01 | 0.002 |
| Shriveling (score) | - | - | 0.21 | < 0.001 |
|  |  |  |  |  |
