# Supplementary material for: Understanding the genetic basis of blueberry postharvest traits to define better breeding strategies
Source: G3 (Bethesda). 2024 Jul 25;14(9):jkae163. doi: 10.1093/g3journal/jkae163 (PMC11373639; doi:10.1093/g3journal/jkae163)
Supplement: jkae163_Supplementary_Data [file jkae163_supplementary_data.zip › Table_S4_G3-2024-405222.docx]

**Table S4.** Genetic correlations (ρ) estimated using restricted maximum likelihood (REML) for each pair of postharvest time points in the longitudinal models.

| Trait | ρ_1d1w_ | ρ_1d3w_ | ρ_1d7w_ | ρ_1w3w_ | ρ_1w7w_ | ρ_3w7w_ |
| --- | --- | --- | --- | --- | --- | --- |
| Firmness (g/mm) | 0.957 | 0.854 | 0.610 | 0.945 | 0.746 | 0.841 |
| TTA (%) | 0.999 | 0.998 | 0.984 | 0.999 | 0.999 | 0.968 |
| SSC (brix) | 0.998 | 0.998 | 0.985 | 0.999 | 0.996 | 0.979 |
| Size (mm) | 0.999 | 0.999 | 0.957 | 0.999 | 0.956 | 0.986 |
| Bloom (visual score) | 0.881 | 0.655 | 0.896 | 0.733 | 0.873 | 0.999 |
| ΔFirmness (g/mm) | - | - | - | 0.465 | 0.218 | 0.581 |
| ΔTTA (%) | - | - | - | 0.513 | 0.448 | 0.666 |
| Shriveling (score) | - | - | - | 0.249 | 0.142 | 0.217 |
